# Have China’s internal migrants been more settled since 2010? A contribution based on migrants’ age profiles

**DOI:** 10.1080/15387216.2024.2365893

**Published:** 2024-06-20

**Authors:** Xiaxia Yang, Kam Wing Chan

**Affiliations:** a Lau China Institute, King’s College London, London, UK; b Department of Geography, University of Washington, Seattle, WA, USA

**Keywords:** Age, migrant settlement, *hukou system*, China, migrant stock

## Abstract

This Research Note employs age data to assess whether China’s internal migrants have become more settled since 2010. The aim is to answer the crucial question of whether recent *hukou* reform initiatives have achieved their goals of improving migrant settlement. We extend a method from existing research that examines two key aspects of settlement – family togetherness and long-term stay of migrants – through analyzing age profiles of migrant stock. Specifically, by scrutinizing age data from the 2010 and 2020 censuses, we evaluate whether the child and elderly dependents of migrants are more engaged in migration and whether migrants growing old can better remain in the destinations. The results show that from 2010 to 2020, the age distribution of migrants became slightly more even across all age groups, yet it was still concentrated in young adults – the overall shape remained largely unchanged. This indicates a small improvement in settlement, which is unsatisfactory given the various *hukou* reform initiatives aimed at substantially increasing settlement opportunities in the last 10 years. Much greater efforts on *hukou* reform and support for migrants are needed. Furthermore, our research highlights the utility of age data in assessing the actual extent of migrant settlement, as opposed to the exclusive focus on settlement intentions, which are common in existing studies.

## Introduction

Over the Lunar New Year, one anticipates witnessing a mass migration across China. During this weeks-long holiday, hundreds of millions of people undertake long-distance travel to go home. This annual spectacle has occurred since the mid-1980s, with exceptions in 2021 and 2022 due to stringent Covid-related lockdowns. The Lunar New Year interlude serves as a rare occasion for hundreds of millions of internal migrant families to be reunited, as, during the rest of the year, members of these families are often split up in different locales. In 2020, nearly 90 million children of internal migrants, predominantly in rural areas, did not live with both parents (Chan 2023b). This phenomenon highlights the enormous challenges migrants and their families face in settling in the destination cities, a distinctive feature of China’s migration regime. Extensive literature on China’s *hukou* system elucidates its role in hindering rural-to-urban migrant workers from establishing permanent residency in the cities. Many young workers who migrate to the cities find it difficult to stay as they grow old and have to return to their origins. At the same time, their dependents, especially children, face major institutional barriers and other challenges to migrate together with the migrant workers (Guo 2014). China’s renowned late sociologist Lu Xueyi (2005) has pointedly said that this model extracts the healthy youth labor from the rural areas but leaves the elderly and the sick in the countryside.

Distinguishing between migrant flow and stock, Yang and Chan (2023) used age data to demonstrate the difficulties migrants face when trying to permanently settle in the destination cities. This research builds upon but extends their work to compare the age profiles of migrant stock in different years, instead of comparing those of the flow and stock. Using age data, we also leverage a precious opportunity to empirically assess whether China’s internal migrants were more settled in 2020 than in 2010. The latest 2020 census has publicly released age data for the “floating population” (FP, *liudong renkou* 流动人口), which is a stock concept, enabling a comparison with the 2010 census data – the only other instance where age data for FP were publicly available in China’s census history dating back to 1953.

In this Research Note, we conduct an analysis of the age profiles of migrant stock to determine if China’s internal migrants have become more settled since 2010—an essential inquiry given China’s imperative need to reform its *hukou *system, which has contributed to a two-class Chinese society (Chan and Wei 2019; Whyte 2010). Addressing this issue is crucial for fostering conditions that allow more migrants, including migrant children, to settle in cities and attend school (Lu 2016). Scholars have also argued that promoting the settlement of migrants is pivotal in invigorating China’s domestic consumption (Cai 2020). Although China has pledged *hukou* reform for decades without substantial success (Chan 2021; Chan and Buckingham 2008), the early 2010s witnessed a notable escalation in the *hukou* reform initiatives, culminating in the ambitious “New-type National Urbanization Plan, 2014–2020” (Chan 2014). The 2014 plan was aimed at settling 100 million migrants in cities and reducing the percentage of the FP. Some have even argued that China entered a new phase of urbanization in the 2010s with an expected acceleration of migrants’ settlement (Lin and Zhu 2022).

Since the 2010s, numerous studies on China’s internal migration have focused on migrants’ *intentions* to settle, not their *actual* settlement (e.g. Fan 2011; Sheng and Zhao 2021). Settlement is a complex process typically involving gaining resident status, employment, acculturation, and other factors (Tan 2016). Settlement intentions are often loose plans, influenced by many uncertainties out of the control of migrants and their families (Chan 2023a). The current exercise diverges by examining the *actual* settlement outcomes through the lens of age. Also, we use data from full national censuses rather than small-scale sample surveys, as the intention research does. Following Yang and Chan (2023), this research pays special attention to these two key aspects of settlement: family togetherness and long-term stay of migrants. It contributes to the limited body of work assessing the progress of recent *hukou* reform, which uses indicators such as the “two rates” of urbanization (*lianglv zhicha* 两率之差) or the size and percentage of the “left-behind children” (Chan 2023b; Li and Zhang 2021).

We begin by outlining the core concepts in Yang and Chan’s work (2023), and then explain a method for using age data of migrant stock over time to gauge the changing degree of migrant settlement. We normalize the age profiles of China’s 2010 and 2020 migrant stock to separate the impact of migrant settlement on age profiles. We finally discuss the results in conjunction with other relevant studies and pinpoint the significance of this research.

## Migrant settlement and age profiles

Yang and Chan (2023) developed a conceptual framework that draws on age profiles of migrant flow and stock to study migrant settlement. Migrant flow is the population who migrate within a certain time period, commonly ranging from one to five years, whereas migrant stock is the remaining (existing) migrants at a certain time point, accumulated from past waves of migration. Typically, migrant flow is dominated by young adults and, to a lesser extent, their child dependents, whereas migrant stock has a relatively smooth age distribution. The stock comprises flows from successive age cohorts who arrive at different times and remain in the destination, including both early and recent cohorts. For recent migrant cohorts, their age profile aligns with that of the flow, with concentrations of young adults and children. For early migrant cohorts, they have lived in the destination for a longer time and have become older. As time passes, dependents of the early migrants are brought from their origins and reunited with them at the destinations. Over time, the age profile of the flow, characterized by peaks at young adults’ and children’s ages, gradually transits to a stock profile that distributes much more evenly across all age groups, as a result of the aging of these early cohorts and the arrival of their dependents.

**Figure 1.**
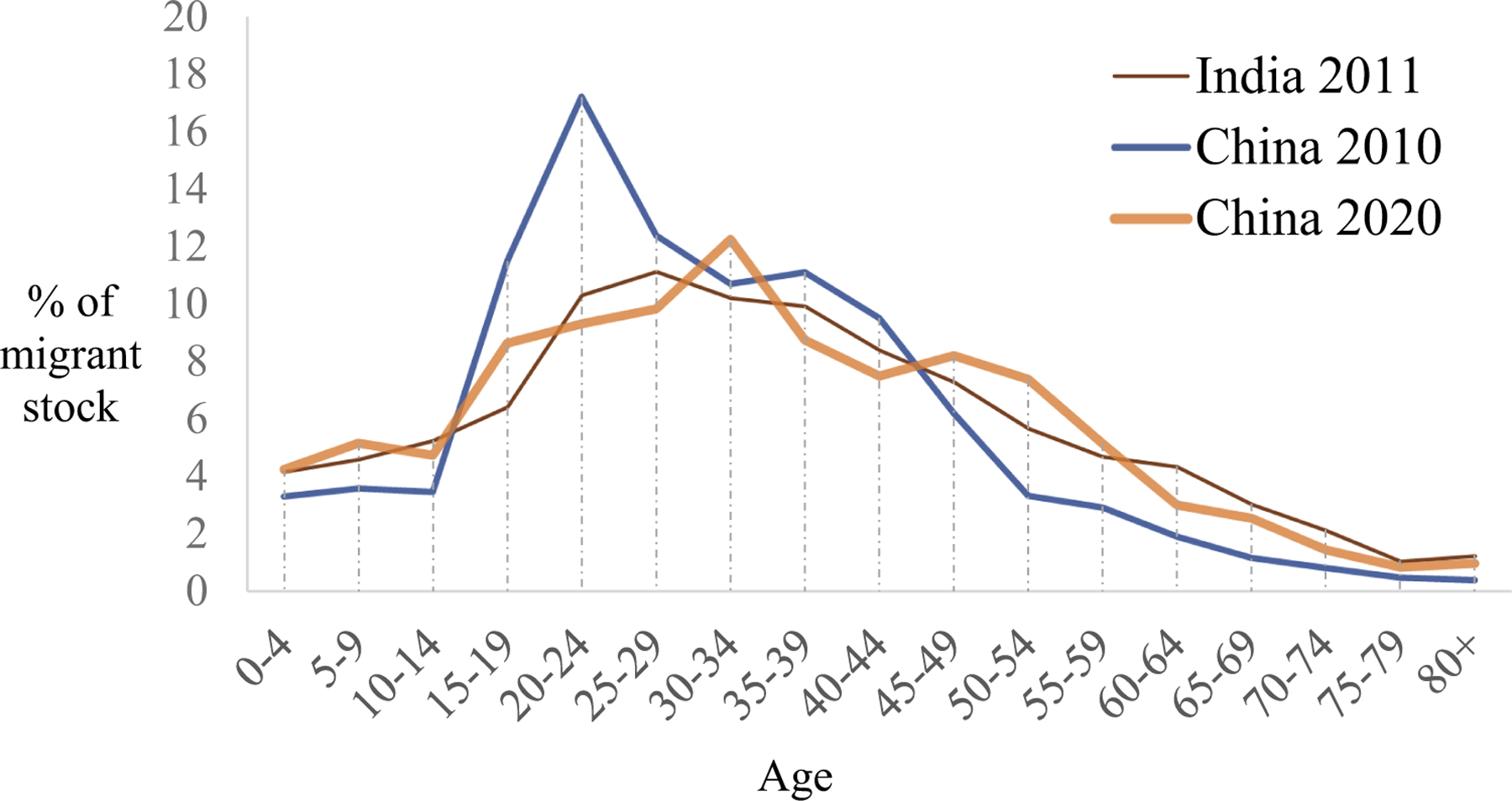
Age structure of migrant stock of India in 2011 and China in 2010 and 2020. Sources: RGCCI (2011); NBS (2012, 2022).

Yang and Chan (2023) used the age profile of India’s migrant stock to show the typical age profile of a country with no significant institutional barriers to internal migration and settlement. As shown in Figure 1, India’s migrant stock age profile is considerably smoother than China’s. Besides being smoother, India’s stock is also older than China’s. With migrant flow serving as the benchmark, the difference between the flow and stock median ages is about 9 years in India but only 2.7 years in China (Yang and Chan 2023). In short, for a migrant population that does not face serious migration and settlement barriers, its stock typically has a smoother and older age profile.

Unlike many other countries in the world like India, China has strong institutional barriers that impede internal migration and their eventual settlement. Most notable among them are the *hukou* (户口) system and its various point-based variants, such as the residence permit (*juzhuzheng*居住证) and the eligibility criteria for school enrollment. The *hukou* system categorizes individuals as either “locals” (*hukou* population) or “non-locals” (non-*hukou* population or FP) based on their household registration place, granting full access to urban social benefits to local *hukou* holders only, including education for their children. The FP legally does not have access to those benefits, but in recent years, many cities have extended limited benefits to some to help them settle, though several are mostly “phantom services” (Chan and O’Brien 2019).

## Settlement trends of China’s non-*hukou* migrant stock, 2010–2020

Using the analytical approach outlined above, one can compare the age profiles of migrant stock over time in the same country to identify the changing settlement trends. A smoother and older age profile of the stock would indicate that the migrant population is more settled, though it is also possibly a result of population aging of the whole country. Thus, to sort out the settlement effect, one also needs to isolate the influence of the age structure of the population from whom migrants originate, i.e. the “at-risk” population of migration (ARP).

**Figure 2.**
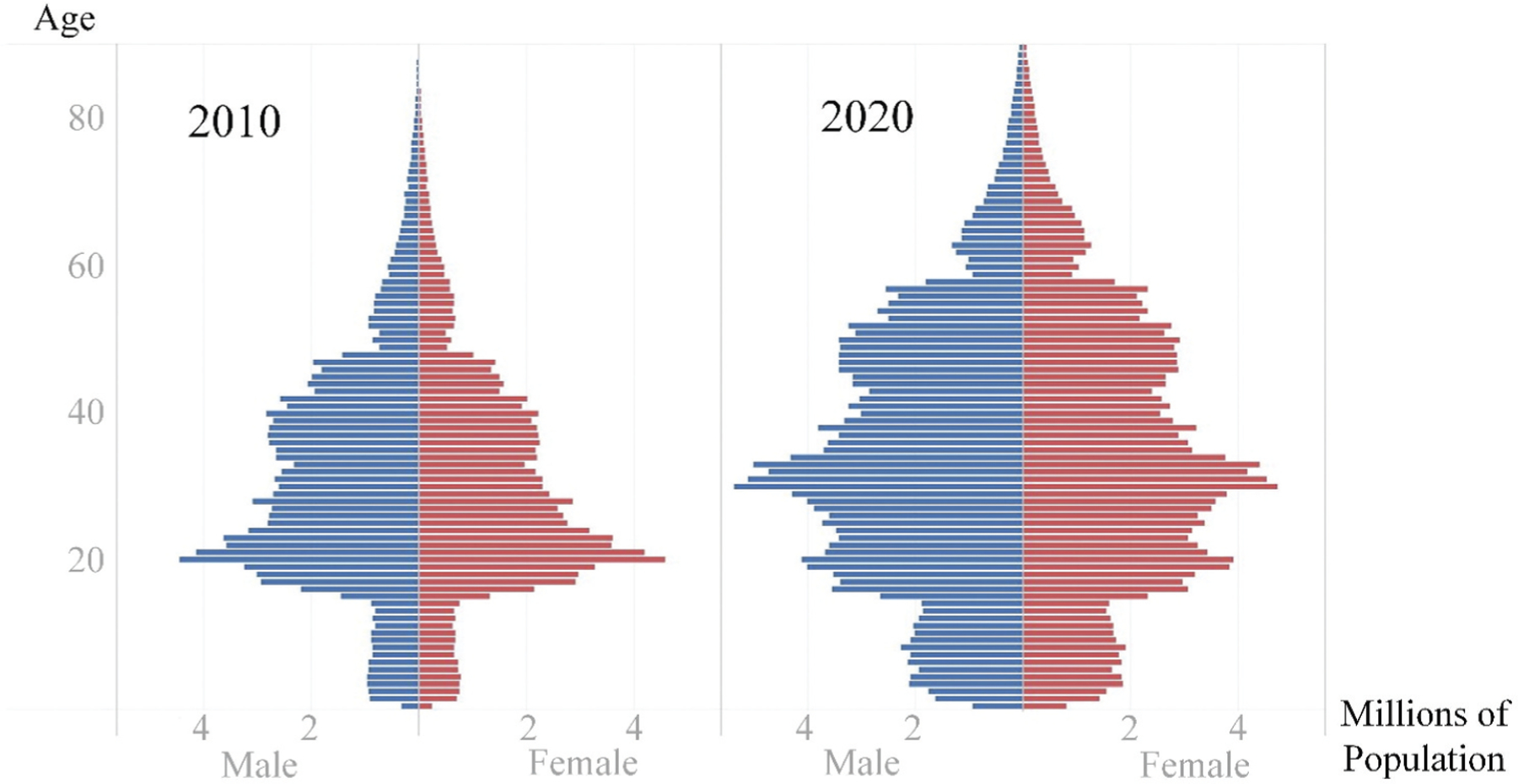
Age pyramid of the “floating population” in China, 2010 and 2020. Sources: NBS (2012, 2022).

In the following, we examine the age composition of China’s migrant stock, the FP, in 2010 and 2020. In this 10-year period, the urban population rose by 232 million; the FP increased by 155 million, to 376 million in 2020 (NBS 2012, 2022). Figure 2 displays the age pyramids of the FP, showing a shift in the shape from a narrowly based “Christmas tree” in 2010, when young adults in their twenties made up the largest group, to a multi-layered “pagoda” in 2020, when working-age adults between 20 and 50 formed the majority. This is consistent with what is displayed in Figure 1—the smoother age curve in 2020 suggests that the extent of concentration in young adults has become less pronounced over the decade. Moreover, the modal age group shifted from 20–24 to 30–34 (Figure 1), suggesting that the FP became older than before. The median age also moved from 29.38 to 33.19 (Table 1), an increase of 3.81 years.

**Figure 3.**
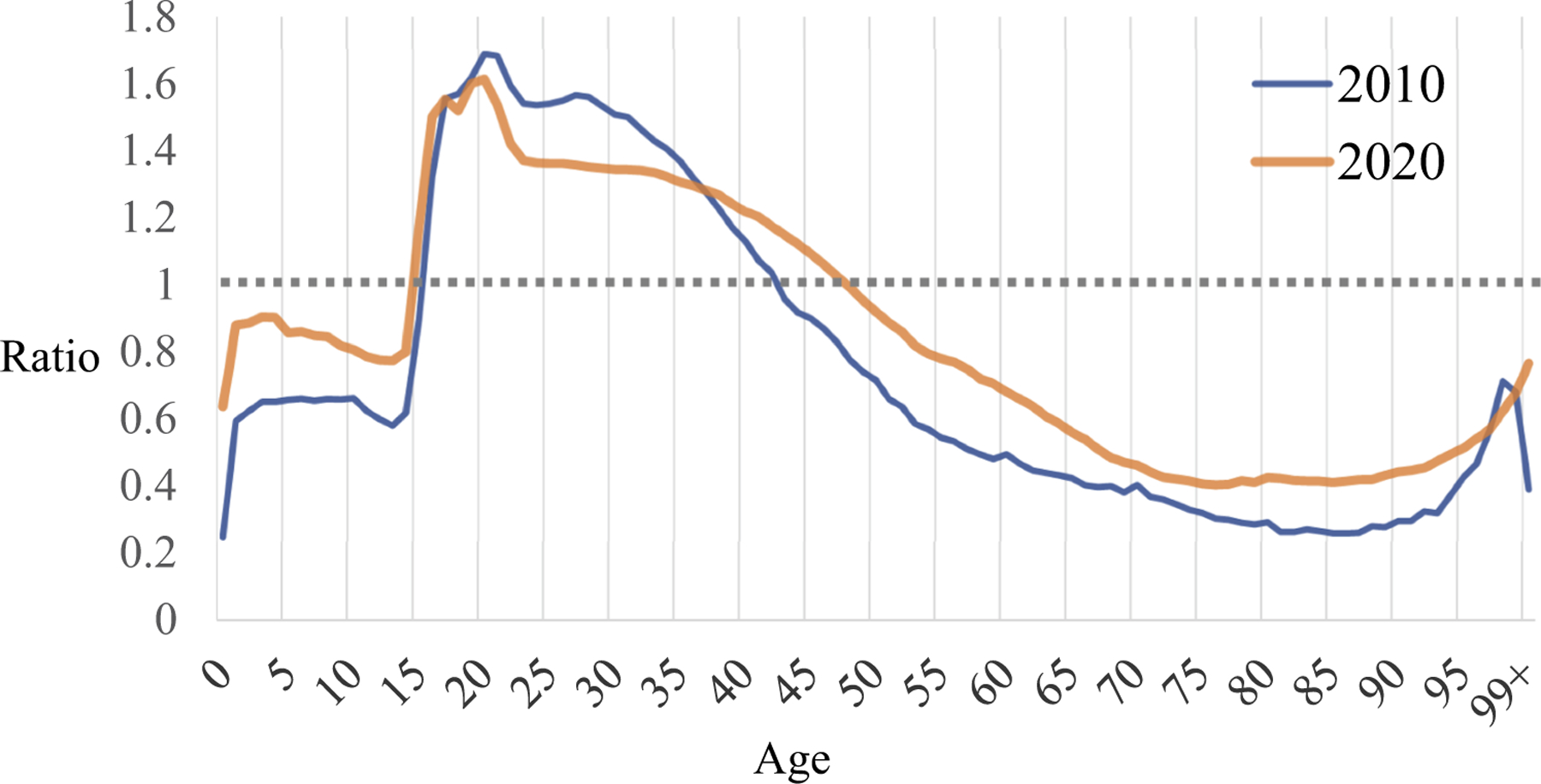
Ratio of the “floating population” to “at-risk population” in China by age, 2010 and 2020. Sources: NBS (2012, 2022).

As pointed out above, the aging of the FP could also result from the general aging of the Chinese population. To control for that effect, we compare the change in age patterns of the total population and, even better, the ARP, from which the pool of migrants is drawn. We use the sum of the *de facto* rural population and the existing migrant stock as a proxy of the ARP because the great majority of the FP are from rural areas (Zhou 2021). The results of these normalizations are shown in Table 1 and Figure 3.

From 2010 to 2020, the difference in the median age between total population and FP decreased from 6.55 to 6.23; that between ARP and FP, barely changed from 4.93 to 4.92 (Table 1). This indicates that there is likely a small improvement in migrant settlement. To further pinpoint the specific changes, we plot the ratio of the percentage of each age group’s FP and ARP in Figure 3. This is to normalize the former by the latter and to present the over- or under-concentration of migrant age groups. A ratio of 1, shown by the horizontal dotted line in Figure 3, indicates that the percentage of FP in a certain age group is the same as the percentage of ARP in that age group. Thus, any deviation from 1 would suggest the age selectivity of FP relative to ARP. As expected, the ratio curves show that the FP was under-represented above age 43 in 2010 or 48 in 2020, as well as below 16 in 2010 or 15 in 2020. The under-representation was especially serious above 55. Conversely, the FP was over-represented in age 15–43 in 2010 or 16–48 in 2020. The two curves look largely the same despite the increase of 70% of FP in the decade. There is some progress in settlement as the 2020 curve is smoother, closer to the dotted line of ratio 1. The result indicates that child and elderly migrants are slightly better able to migrate and stay, and that earlier migrants have a bigger chance to remain in the destinations rather than leave for their origins when growing older. Considering findings reported in the literature (Chan 2023a), it becomes clear that these improvements primarily took place in lower-tier cities and towns, while settling in first-tier cities remained challenging for most migrants.

## Conclusion

To summarize our findings, based on analyses of age structure, modal age, median age, and the ratio of FP to ARP by age, we have shown that China’s internal migrants are slightly more settled in 2020 than in 2010. However, the fundamental configuration remains largely unchanged. The situation is particularly unsatisfactory given the government’s loud pronouncements and commitments to the *hukou* reform since the early 2010s. Our observation aligns with other research that evaluates recent major *hukou* reform initiatives. For instance, Li and Zhang (2021) and Chan (2023a) revealed that although China successfully converted 100 million rural *hukou* holders to urban *hukou* holders between 2014 and 2020, it failed to reduce the percentage of rural *hukou* holders in urban area, a crucial benchmark as planned. Moreover, in a study on China’s children of migrants, Chan (2023b) found that the size of the “left-behind children” significantly increased from 68 million in 2010 to 89 million in 2020. In 2020, the “left-behind children” accounted for 30% of all children in China, up from 24% in 2010. Our research underscores the urgent need for substantial reform of the *hukou* system to support the settlement of migrants and their families, especially in light of China’s lackluster post-Covid economic recovery in 2023, as emphasized by scholars like Cai (2024). Indeed, China should seriously consider phasing out the *hukou* system altogether instead of merely “reforming” it, as argued by Chan (2023a). This institution has presented numerous fundamental challenges to China’s path toward establishing an inclusive, human-centered single society, and these problems cannot be easily remedied without fundamental change to the *hukou* system. Equally noteworthy in this study is the demonstration that age data, easily derivable from publicly available sources such as national and local population tabulations, can contribute to gauging migrant settlement, whether in China or other countries and cities worldwide. Our research is grounded in full census data and measures actual settlement outcomes, as opposed to many other studies that focus on settlement intentions.

## Figures and Tables

**Table 1.** Median age of the total population, “at-risk population”, and “floating population” in China.

		2010			2020		
Population group		Total	ARP	FP	Total	ARP	FP
Median age		35.93	34.31	29.38	39.42	38.11	33.19
Difference	Total and FP		6.55			6.23	
	ARP and FP		4.93			4.92	

Notes: “Total”, “ARP”, and “FP” refer to the total, at-risk, and floating population. Sources: NBS (2012, 2022).
